# *N*-Cyclo­pentyl-*N*-(3-oxo-2,3-dihydro-1*H*-inden-1-yl)acetamide

**DOI:** 10.1107/S160053681200606X

**Published:** 2012-03-03

**Authors:** Tao Zhang, Tom McCabe, Bartosz Marzec, Neil Frankish, Helen Sheridan

**Affiliations:** a Drug Discovery Group, School of Pharmacy and Pharmaceutical Sciences, Trinity College Dublin, Dublin 2, Ireland; b Trino Therapeutics Ltd, Unit 2.5 The Tower, Trinity Technology & Enterprise Campus, Pearse Street, Dublin 2, Ireland; c School of Chemistry, Trinity College Dublin, Dublin 2, Ireland

## Abstract

The title mol­ecule, C_16_H_19_NO_2_, consists of an indane moiety, which is connected through an N atom to an acetamide group and a cyclo­pentane ring. The N atom adopts planar triangular geometry. Inter­molecular inter­actions, such as π–π stacking or hydrogen bonding, were not observed.

## Related literature

For background information on the indane pharmacophore, see: Vaccva *et al.* (1994 ▶); Buckle *et al.* (1973 ▶); Heinzelmann *et al.* (1940 ▶). For details of the pharmacological activity of the title compound, see: Sheridan *et al.* (1990 ▶, 1999*a* ▶,*b* ▶, 2008 ▶); Frankish *et al.* (2004 ▶). For ionization characteristics, see: Simplício *et al.* (2004 ▶).
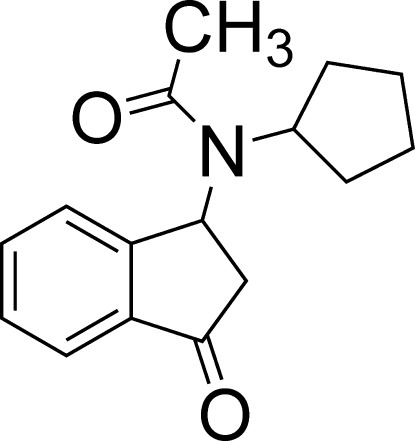


## Experimental

### 

#### Crystal data

C_16_H_19_NO_2_
*M* *_r_* = 257.32
Triclinic, 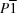

*a* = 8.1539 (16) Å
*b* = 8.9944 (18) Å
*c* = 10.084 (2) Å
α = 87.97 (3)°
β = 81.29 (3)°
γ = 63.15 (3)°
*V* = 651.8 (2) Å^3^
*Z* = 2
Mo *K*α radiation
μ = 0.09 mm^−1^
*T* = 150 K
0.60 × 0.50 × 0.30 mm

#### Data collection

Rigaku Saturn 724 diffractometer
Absorption correction: multi-scan (*CrystalClear*; Rigaku, 2006 ▶) *T*_min_ = 0.726, *T*_max_ = 1.000
7260 measured reflections
2191 independent reflections
2157 reflections with *I* > 2σ(*I*)
*R*_int_ = 0.026

#### Refinement

*R*[*F* ^2^ > 2σ(*F* ^2^)] = 0.042
*wR*(*F* ^2^) = 0.100
*S* = 1.13
2191 reflections
174 parameters
H-atom parameters constrained
Δρ_max_ = 0.21 e Å^−3^
Δρ_min_ = −0.23 e Å^−3^

### 

Data collection: *CrystalClear* (Rigaku, 2006 ▶); cell refinement: *CrystalClear*; data reduction: *CrystalClear*; program(s) used to solve structure: *SHELXS97* (Sheldrick, 2008 ▶); program(s) used to refine structure: *SHELXL97* (Sheldrick, 2008 ▶); molecular graphics: *DIAMOND* (Brandenburg, 1998 ▶); software used to prepare material for publication: *SHELXL97*.

## Supplementary Material

Crystal structure: contains datablock(s) I, global. DOI: 10.1107/S160053681200606X/hg5169sup1.cif

Structure factors: contains datablock(s) I. DOI: 10.1107/S160053681200606X/hg5169Isup2.hkl

Supplementary material file. DOI: 10.1107/S160053681200606X/hg5169Isup3.cml

Additional supplementary materials:  crystallographic information; 3D view; checkCIF report

## Figures and Tables

**Fig. 1.**
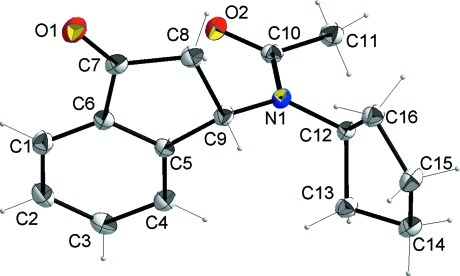
The molecular structure of the title compound with the atom numbering scheme. Displacement ellipsoids are drawn at the 50% probability level. H atoms are presented as small spheres of arbitrary radius.

**Fig. 2.**
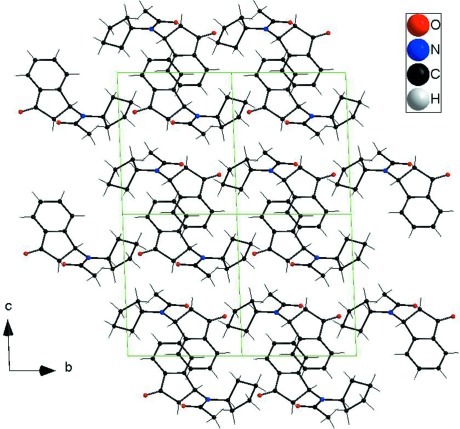
Packing diagram of the title compound viewed along the crystallographic a-axis.

## supplementary crystallographic information

### Comment

The indane pharmacophore occurs in many different bioactive molecules. Indinavir, a HIV-1 inhibitor is a protease inhibitor in clinical use that contains an indane fragment (Vaccva *et al.*, 1994). Nivemedone, a nitro-indanone has anti-allergenic activity (Buckle *et al.*, 1973) while many simple indanols demonstrate bronchodilatory activity (Heinzelmann *et al.*, 1940). We have demonstrated that indanone derivatives possess smooth muscle relaxant activity and inhibit mediator release (Sheridan *et al.*, 1990, 1999*a*, 1999*b*; Frankish *et al.*, 2004). In a recent study on bioactivity we evaluated the smooth muscle relaxant activity and mediator release inhibition activities demonstrated by a series of aminoindanones (Simplício *et al.*, 2004; Sheridan *et al.*, 2008).

The asymmetric unit of the compound presented in this paper contains a single molecule of *N*-cyclopentyl-*N*-(3-oxo-2,3-dihydro-1*H* -inden-1-yl)acetamide. The geometry around the nitrogen atom can be best described as trigonal planar. As there are no flexible hydrogen atoms attached to the nitrogen atom N1 or the oxygen atoms (O1 and O2) hydrogen bonding do not prevail in the title compound. The shortest distance between the aromatic rings is 4.150 (9) Å and cannot be considered as the π-π stacking interaction.

The packing diagram of the structure, presented in Fig. 2, shows that the molecules are separated and when viewed along the crystallographic *a*-axis seem to form a sheet-like structure in the *ab*-plane. These sheets pack in the direction of the crystallographic *c*-axis. The shortest separation distance between them is 4.243 (75) Å and a weak Van der Vaals force or an electrostatic interaction may be responsible for holding the sheets together.

### Experimental

The title compound was synthesized as reported (Sheridan *et al.*, 2008). *N*-Bromosuccinimide (672 mg, 3.78 mmol) and a catalytic amount of dibenzoylperoxide were added to a solution of indan-1-one (500 mg, 3.78 mmol) in CCl_4_ (15 ml) and the reaction was refluxed for 45 min. After cooling, the reaction was washed with water, dried over Na_2_SO_4_, filtered and evaporated *in vacuo*. The resultant was purified by column chromatography over silica gel (eluant, pet. ether:EtOAc, 4:1) to yield 3-bromoindan-1-one as an oil. To this 3-bromoindan-1-one solution (200 mg, 0.95 mmol) in dry DCM was added cyclopentanamine (80 mg, 0.94 mmol) and triethylamine (200 mg, 1.98 mmol). The reaction was stirred at 0°C for 3 h. The solvent was removed *in vacuo* and the residue was purified directly by flash column chromatography on silica gel (eluant, pet. ether:EtOAc, 4:1). After evaporation of the eluent the secondary amine was isolated as an oil (175 mg, 86%). To this secondary amine solution (700 mg, 3.25 mmol) in DCM (5 ml) was added triethylamine (657 mg, 0.90 ml, 6.51 mmol), acetic anhydride (664 mg, 0.61 ml, 6.51 mmol) and DMAP (476 mg, 3.90 mmol). The reaction was stirred at room temperature for 2 h. The reaction mixture was then washed with water, dried over Na_2_SO_4_, filtered and evaporated *in vacuo*. The residue was purified by column chromatography over silica gel (pet. ether:EtOAc, 4:1) to yield the title compound as a white solid (450 mg, 54%). Crystals suitable for X-ray diffraction were obtained after 5 days of slow evaporation of an ethanol solution.

### Refinement

All H atoms were placed in geometrically idealized positions and constrained to ride on their parent atoms with C—H = 0.93 Å for aromatic H atoms, 0.96 Å for CH_3_ type H atoms, 0.97 Å for CH_2_ type H atoms and 0.98 Å for CH type H atoms, respectively. *U*_iso_(H) values were set at 1.5*U*_eq_(C) for methyl H atoms, and 1.2*U*_eq_(C)for the rest of the H atoms.

### Crystal data

**Table:**

C_16_H_19_NO_2_	*Z* = 2
*M**_r_* = 257.32	*F*(000) = 276
Triclinic, *P*1	*D*_x_ = 1.311 Mg m^−^^3^
Hall symbol: -P 1	Mo *K*α radiation, λ = 0.71073 Å
*a* = 8.1539 (16) Å	Cell parameters from 2546 reflections
*b* = 8.9944 (18) Å	θ = 2.0–31.2°
*c* = 10.084 (2) Å	µ = 0.09 mm^−^^1^
α = 87.97 (3)°	*T* = 150 K
β = 81.29 (3)°	Prism, colourless
γ = 63.15 (3)°	0.60 × 0.50 × 0.30 mm
*V* = 651.8 (2) Å^3^	

### Data collection

**Table:**

Rigaku Saturn 724 diffractometer	2191 independent reflections
Radiation source: fine-focus sealed tube	2157 reflections with *I* > 2σ(*I*)
Graphite monochromator	*R*_int_ = 0.026
Detector resolution: 28.5714 pixels mm^-1^	θ_max_ = 25.0°, θ_min_ = 2.8°
ω and phi scans	*h* = −9→9
Absorption correction: multi-scan (*CrystalClear*; Rigaku, 2006)	*k* = −10→8
*T*_min_ = 0.726, *T*_max_ = 1.000	*l* = −11→11
7260 measured reflections	

### Refinement

**Table:**

Refinement on *F*^2^	Primary atom site location: structure-invariant direct methods
Least-squares matrix: full	Secondary atom site location: difference Fourier map
*R*[*F*^2^ > 2σ(*F*^2^)] = 0.042	Hydrogen site location: inferred from neighbouring sites
*wR*(*F*^2^) = 0.100	H-atom parameters constrained
*S* = 1.13	*w* = 1/[σ^2^(*F*_o_^2^) + (0.0406*P*)^2^ + 0.3106*P*] where *P* = (*F*_o_^2^ + 2*F*_c_^2^)/3
2191 reflections	(Δ/σ)_max_ < 0.001
174 parameters	Δρ_max_ = 0.21 e Å^−^^3^
0 restraints	Δρ_min_ = −0.23 e Å^−^^3^

### Special details

**Table:**

Experimental. The su's on the Cell Angles were measured.
Geometry. All e.s.d.'s (except the e.s.d. in the dihedral angle between two l.s. planes) are estimated using the full covariance matrix. The cell e.s.d.'s are taken into account individually in the estimation of e.s.d.'s in distances, angles and torsion angles; correlations between e.s.d.'s in cell parameters are only used when they are defined by crystal symmetry. An approximate (isotropic) treatment of cell e.s.d.'s is used for estimating e.s.d.'s involving l.s. planes.
Refinement. Refinement of *F*^2^ against ALL reflections. The weighted *R*-factor *wR* and goodness of fit *S* are based on *F*^2^, conventional *R*-factors *R* are based on *F*, with *F* set to zero for negative *F*^2^. The threshold expression of *F*^2^ > σ(*F*^2^) is used only for calculating *R*-factors(gt) *etc*. and is not relevant to the choice of reflections for refinement. *R*-factors based on *F*^2^ are statistically about twice as large as those based on *F*, and *R*- factors based on ALL data will be even larger.

### Fractional atomic coordinates and isotropic or equivalent isotropic displacement parameters (Å^2^)

**Table:**

	*x*	*y*	*z*	*U*_iso_*/*U*_eq_	
O2	0.31603 (15)	0.52993 (14)	0.35282 (11)	0.0247 (3)	
N1	0.26193 (16)	0.31399 (15)	0.30730 (12)	0.0180 (3)	
C12	0.3026 (2)	0.13741 (18)	0.31299 (14)	0.0178 (3)	
H12	0.4075	0.0812	0.3627	0.021*	
C5	0.15437 (19)	0.51234 (18)	0.12014 (14)	0.0178 (3)	
C9	0.10493 (19)	0.42530 (18)	0.24012 (14)	0.0182 (3)	
H9	0.0535	0.3566	0.2071	0.022*	
C6	0.0277 (2)	0.67950 (19)	0.12237 (14)	0.0188 (3)	
C10	0.3582 (2)	0.38022 (19)	0.36131 (14)	0.0191 (3)	
C4	0.2976 (2)	0.4435 (2)	0.01256 (15)	0.0221 (3)	
H4	0.3821	0.3311	0.0095	0.027*	
C7	−0.1091 (2)	0.72363 (19)	0.24640 (15)	0.0199 (3)	
C11	0.5168 (2)	0.2668 (2)	0.43334 (16)	0.0245 (4)	
H11A	0.5711	0.3301	0.4669	0.037*	
H11B	0.6092	0.1799	0.3720	0.037*	
H11C	0.4709	0.2182	0.5069	0.037*	
C1	0.0406 (2)	0.7826 (2)	0.01944 (15)	0.0226 (3)	
H1	−0.0454	0.8945	0.0219	0.027*	
C16	0.1404 (2)	0.10622 (19)	0.38594 (15)	0.0222 (3)	
H16A	0.1517	0.0863	0.4801	0.027*	
H16B	0.0218	0.2016	0.3793	0.027*	
C13	0.3576 (2)	0.04558 (19)	0.17613 (14)	0.0205 (3)	
H13A	0.2763	0.1118	0.1132	0.025*	
H13B	0.4852	0.0175	0.1386	0.025*	
C3	0.3120 (2)	0.5459 (2)	−0.09033 (15)	0.0245 (4)	
H3	0.4079	0.5014	−0.1624	0.029*	
C14	0.3337 (2)	−0.11046 (19)	0.21048 (15)	0.0227 (3)	
H14A	0.4397	−0.1927	0.2482	0.027*	
H14B	0.3200	−0.1595	0.1313	0.027*	
C2	0.1849 (2)	0.7143 (2)	−0.08713 (15)	0.0251 (4)	
H2	0.1969	0.7811	−0.1566	0.030*	
C8	−0.0572 (2)	0.56929 (19)	0.32894 (15)	0.0223 (3)	
H8A	−0.1624	0.5451	0.3517	0.027*	
H8B	−0.0181	0.5849	0.4113	0.027*	
C15	0.1556 (2)	−0.04907 (19)	0.31440 (16)	0.0242 (4)	
H15A	0.1640	−0.1346	0.3782	0.029*	
H15B	0.0478	−0.0206	0.2704	0.029*	
O1	−0.24001 (15)	0.85977 (14)	0.27869 (11)	0.0280 (3)	

### Atomic displacement parameters (Å^2^)

**Table:**

	*U*^11^	*U*^22^	*U*^33^	*U*^12^	*U*^13^	*U*^23^
O2	0.0310 (6)	0.0181 (7)	0.0266 (6)	−0.0122 (5)	−0.0055 (5)	0.0010 (4)
N1	0.0186 (6)	0.0145 (7)	0.0187 (6)	−0.0058 (5)	−0.0023 (5)	0.0003 (5)
C12	0.0196 (7)	0.0130 (8)	0.0187 (7)	−0.0058 (6)	−0.0016 (6)	0.0001 (6)
C5	0.0174 (7)	0.0187 (8)	0.0181 (7)	−0.0083 (6)	−0.0052 (5)	0.0009 (6)
C9	0.0168 (7)	0.0159 (8)	0.0192 (7)	−0.0055 (6)	−0.0011 (5)	−0.0003 (6)
C6	0.0187 (7)	0.0180 (8)	0.0213 (7)	−0.0083 (6)	−0.0072 (6)	0.0007 (6)
C10	0.0206 (7)	0.0199 (9)	0.0154 (7)	−0.0089 (6)	0.0008 (5)	−0.0016 (6)
C4	0.0209 (7)	0.0201 (9)	0.0225 (8)	−0.0071 (6)	−0.0021 (6)	−0.0006 (6)
C7	0.0164 (7)	0.0190 (9)	0.0235 (8)	−0.0063 (6)	−0.0054 (6)	−0.0029 (6)
C11	0.0248 (8)	0.0229 (9)	0.0271 (8)	−0.0108 (7)	−0.0072 (6)	−0.0002 (6)
C1	0.0235 (8)	0.0192 (8)	0.0266 (8)	−0.0096 (6)	−0.0092 (6)	0.0030 (6)
C16	0.0236 (8)	0.0196 (9)	0.0212 (7)	−0.0092 (7)	0.0017 (6)	−0.0008 (6)
C13	0.0188 (7)	0.0199 (9)	0.0192 (7)	−0.0061 (6)	−0.0007 (6)	−0.0021 (6)
C3	0.0239 (8)	0.0313 (10)	0.0192 (8)	−0.0136 (7)	−0.0019 (6)	0.0000 (6)
C14	0.0220 (8)	0.0186 (9)	0.0255 (8)	−0.0071 (7)	−0.0032 (6)	−0.0044 (6)
C2	0.0306 (8)	0.0299 (10)	0.0211 (8)	−0.0181 (8)	−0.0091 (6)	0.0079 (6)
C8	0.0189 (7)	0.0206 (9)	0.0218 (8)	−0.0051 (6)	0.0008 (6)	−0.0015 (6)
C15	0.0235 (8)	0.0183 (9)	0.0304 (8)	−0.0098 (7)	−0.0010 (6)	−0.0012 (6)
O1	0.0219 (6)	0.0211 (7)	0.0327 (6)	−0.0026 (5)	−0.0025 (5)	−0.0031 (5)

### Geometric parameters (Å, º)

**Table:**

O2—C10	1.2334 (19)	C11—H11B	0.9600
N1—C10	1.3571 (19)	C11—H11C	0.9600
N1—C9	1.4687 (19)	C1—C2	1.389 (2)
N1—C12	1.470 (2)	C1—H1	0.9300
C12—C13	1.532 (2)	C16—C15	1.541 (2)
C12—C16	1.548 (2)	C16—H16A	0.9700
C12—H12	0.9800	C16—H16B	0.9700
C5—C6	1.386 (2)	C13—C14	1.523 (2)
C5—C4	1.391 (2)	C13—H13A	0.9700
C5—C9	1.520 (2)	C13—H13B	0.9700
C9—C8	1.551 (2)	C3—C2	1.394 (2)
C9—H9	0.9800	C3—H3	0.9300
C6—C1	1.391 (2)	C14—C15	1.539 (2)
C6—C7	1.476 (2)	C14—H14A	0.9700
C10—C11	1.511 (2)	C14—H14B	0.9700
C4—C3	1.390 (2)	C2—H2	0.9300
C4—H4	0.9300	C8—H8A	0.9700
C7—O1	1.2196 (19)	C8—H8B	0.9700
C7—C8	1.514 (2)	C15—H15A	0.9700
C11—H11A	0.9600	C15—H15B	0.9700
			
C10—N1—C9	118.39 (12)	C6—C1—H1	120.8
C10—N1—C12	124.30 (12)	C15—C16—C12	105.69 (12)
C9—N1—C12	117.30 (12)	C15—C16—H16A	110.6
N1—C12—C13	114.87 (12)	C12—C16—H16A	110.6
N1—C12—C16	114.41 (12)	C15—C16—H16B	110.6
C13—C12—C16	105.21 (12)	C12—C16—H16B	110.6
N1—C12—H12	107.3	H16A—C16—H16B	108.7
C13—C12—H12	107.3	C14—C13—C12	102.50 (12)
C16—C12—H12	107.3	C14—C13—H13A	111.3
C6—C5—C4	120.02 (14)	C12—C13—H13A	111.3
C6—C5—C9	111.41 (13)	C14—C13—H13B	111.3
C4—C5—C9	128.47 (14)	C12—C13—H13B	111.3
N1—C9—C5	115.14 (12)	H13A—C13—H13B	109.2
N1—C9—C8	115.99 (12)	C4—C3—C2	120.98 (15)
C5—C9—C8	103.95 (12)	C4—C3—H3	119.5
N1—C9—H9	107.1	C2—C3—H3	119.5
C5—C9—H9	107.1	C13—C14—C15	104.59 (12)
C8—C9—H9	107.1	C13—C14—H14A	110.8
C5—C6—C1	121.56 (14)	C15—C14—H14A	110.8
C5—C6—C7	110.07 (13)	C13—C14—H14B	110.8
C1—C6—C7	128.36 (14)	C15—C14—H14B	110.8
O2—C10—N1	120.77 (14)	H14A—C14—H14B	108.9
O2—C10—C11	120.75 (13)	C1—C2—C3	120.27 (15)
N1—C10—C11	118.48 (13)	C1—C2—H2	119.9
C3—C4—C5	118.77 (15)	C3—C2—H2	119.9
C3—C4—H4	120.6	C7—C8—C9	106.08 (12)
C5—C4—H4	120.6	C7—C8—H8A	110.5
O1—C7—C6	126.77 (15)	C9—C8—H8A	110.5
O1—C7—C8	125.44 (14)	C7—C8—H8B	110.5
C6—C7—C8	107.79 (13)	C9—C8—H8B	110.5
C10—C11—H11A	109.5	H8A—C8—H8B	108.7
C10—C11—H11B	109.5	C14—C15—C16	105.85 (12)
H11A—C11—H11B	109.5	C14—C15—H15A	110.6
C10—C11—H11C	109.5	C16—C15—H15A	110.6
H11A—C11—H11C	109.5	C14—C15—H15B	110.6
H11B—C11—H11C	109.5	C16—C15—H15B	110.6
C2—C1—C6	118.40 (15)	H15A—C15—H15B	108.7
C2—C1—H1	120.8		
			
C10—N1—C12—C13	−118.48 (15)	C9—C5—C4—C3	176.96 (14)
C9—N1—C12—C13	62.40 (16)	C5—C6—C7—O1	−179.07 (14)
C10—N1—C12—C16	119.67 (15)	C1—C6—C7—O1	2.3 (2)
C9—N1—C12—C16	−59.45 (16)	C5—C6—C7—C8	1.59 (16)
C10—N1—C9—C5	60.63 (17)	C1—C6—C7—C8	−177.04 (14)
C12—N1—C9—C5	−120.20 (14)	C5—C6—C1—C2	−0.1 (2)
C10—N1—C9—C8	−61.05 (17)	C7—C6—C1—C2	178.36 (14)
C12—N1—C9—C8	118.12 (14)	N1—C12—C16—C15	147.25 (12)
C6—C5—C9—N1	−135.65 (13)	C13—C12—C16—C15	20.25 (16)
C4—C5—C9—N1	48.1 (2)	N1—C12—C13—C14	−163.79 (12)
C6—C5—C9—C8	−7.66 (16)	C16—C12—C13—C14	−37.07 (15)
C4—C5—C9—C8	176.08 (14)	C5—C4—C3—C2	−0.6 (2)
C4—C5—C6—C1	−0.7 (2)	C12—C13—C14—C15	39.78 (15)
C9—C5—C6—C1	−177.28 (13)	C6—C1—C2—C3	0.6 (2)
C4—C5—C6—C7	−179.41 (12)	C4—C3—C2—C1	−0.2 (2)
C9—C5—C6—C7	3.98 (16)	O1—C7—C8—C9	174.40 (14)
C9—N1—C10—O2	−0.8 (2)	C6—C7—C8—C9	−6.25 (16)
C12—N1—C10—O2	−179.91 (12)	N1—C9—C8—C7	135.64 (13)
C9—N1—C10—C11	178.57 (12)	C5—C9—C8—C7	8.18 (15)
C12—N1—C10—C11	−0.5 (2)	C13—C14—C15—C16	−27.47 (16)
C6—C5—C4—C3	1.0 (2)	C12—C16—C15—C14	4.32 (16)
